# Awareness of Zoonotic Infections and a Seroprevalence Meta‐Analysis of Brucellosis, Q‐Fever and Toxoplasmosis Among Abattoir Workers

**DOI:** 10.1002/vms3.70985

**Published:** 2026-05-14

**Authors:** Koketso Desiree Mazwi, Charles Byaruhanga, Herman Geyer, Francis Babaman Kolo, Ishmael Festus Jaja, Sunday Ochonu Ochai, Henriette van Heerden

**Affiliations:** ^1^ Department of Veterinary Tropical Diseases Faculty of Veterinary Science University of Pretoria Pretoria South Africa; ^2^ National Agricultural Research Organisation Entebbe Uganda; ^3^ Centre for Emerging, Zoonotic and Parasitic Diseases National Institute for Communicable Diseases National Health Laboratory Services South Africa; ^4^ Department of Animal and Pasture Science Faculty of Science and Agriculture University of Fort Hare Alice South Africa; ^5^ Department of Agriculture and Animal Health University of South Africa Johannesburg South Africa; ^6^ Antimicrobial Research Unit College of Health Sciences University of KwaZulu‐Natal Durban South Africa

**Keywords:** Knowledge, Abattoir, Metadata, Seropositivity, Zoonotic diseases

## Abstract

**Background:**

Workers handling infected animals and carcasses are at risk of zoonotic diseases. However, knowledge, practices and the burden of zoonotic infections among abattoir workers in Africa remain poorly characterized.

**Objectives:**

This study aimed to assess knowledge and practices regarding selected zoonoses among abattoir workers in the Eastern Cape, determine seroprevalence of *Coxiella burnetii* among brucellosis‐seropositive workers in Gauteng, and evaluate variability in seroprevalence of brucellosis, Q‐fever and toxoplasmosis across Africa through a meta‐analysis.

**Methods:**

A cross‐sectional study was conducted among 76 abattoir workers using a structured questionnaire to assess knowledge and practices related to zoonotic diseases. In addition, 92 workers with known brucellosis seropositivity were tested for *C. burnetii* antibodies. A meta‐analysis was conducted to assess the seroprevalence of brucellosis, Q‐fever and toxoplasmosis across African studies.

**Results:**

Univariate analyses revealed that job description and education level were significantly associated (*p* ≤ 0.05) with knowledge of brucellosis and toxoplasmosis. The overall *C. burnetii* seropositivity was 61.9% (95% Cl: 51.3–71.9). A total of 57/92 (61.96%) had IgG antibodies and 1/92 (1.09%) had IgM and IgG antibodies. The meta‐analysis showed that brucellosis seroprevalence ranged from 1.3% to 46.4%, based on two or more tests conducted in 12 African countries. Toxoplasmosis seroprevalence, detected using various single tests, ranged from 2.2% to 84.0% in eight countries, while Q‐fever seroprevalence, assessed in three countries, varied from 6.5% to 37.1%.

**Conclusions:**

This study emphasizes the importance of educating abattoir workers about zoonotic diseases to improve their practices, attitudes and understanding. Abattoir facilities management may improve practices and PPE to encourage workers to follow necessary protocols. The study further demonstrates the limited number of abattoir worker studies on the selected zoonotic diseases observed across Africa, illustrating the importance of further investigations.

## Graphical abstract

Workers handling infected animals and carcasses are at risk of zoonotic diseases. This study emphasizes the importance of educating abattoir workers about zoonotic diseases in order to improve their practices, attitudes and understanding, which can reduce exposure risks. The study further demonstrates the reduced numbers of abattoir worker studies on the selected zoonotic diseases observed across Africa, illustrating the importance of further investigations.

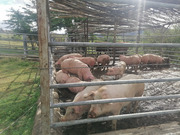

## 1 Introduction

The emergence and re‐emergence of zoonotic diseases reflect an imbalance in human, animal and environmental health, posing a global concern with life‐threatening implications (Woolhouse and Gowtage‐Sequeria 2005). Zoonotic pathogens include parasites, bacteria, viruses or other atypical agents (WHO 2020) that have been reported to constitute 61% of the 1415 known human infectious pathogens, with 75% of zoonoses being emerging or re‐emerging (Taylor et al. 2001). Numerous pathogens, such as *Brucella* spp., *Toxoplasma gondii*, *Chlamydophila* spp., *Coxiella burnetii*, *Listeria* spp. and *Leptospira* spp., that cause abortions in animals have the potential to adversely affect humans (Ganter 2015; Givens and Marley 2008).

Certain occupations, such as farming, veterinary practice, animal husbandry and abattoir work, pose a higher risk of zoonotic disease transmission due to close contact with animals and their products. Abattoir workers, in particular, are exposed to animals and their products during the slaughtering of several species of livestock and are at risk of occupational zoonoses. Brucellosis is a bacterial infection caused by various *Brucella* spp., which poses a threat to both human health and animal productivity (Seleem et al. 2010). Several studies among abattoir workers have been reported from Eastern Africa (Nyamota et al. 2023; Tsegay et al. 2017; Nabukenya et al. 2013) and Central Africa (Awah‐Ndukum et al. 2018; Zein 2014); however, there is no standardized serological test or a combination of tests indicated, thus it is difficult to compare the exposure of abattoir workers among studies.

The intracellular protozoan parasite *T. gondii* is one of the most common human zoonotic pathogens (Montoya 2002) that infects approximately a third of the world population; however, infection rates differ greatly between and even within nations (Saadatnia and Golkar 2012; Pappas et al. 2009). The life cycle of *T. gondii* comprises two phases: a sexual cycle that takes place in the small intestine of the Felidae family, which are the definitive hosts and an asexual cycle in several vertebrate hosts, including humans, which are the intermediate hosts (Black and Boothroyd 2000). In humans, the parasite causes toxoplasmosis, which can be asymptomatic in immunocompetent hosts or present as a moderate nonspecific infection, but can cause abortion in pregnant women (Montoya 2002). Abattoir workers are considered a high‐risk group.

The disease Q‐fever caused by the bacterium *C. burnetii* was first reported in 1935 in Australia after an outbreak among slaughterhouse workers (Derrick 1937), but with fewer publications in South Africa (De Boni et al. 2022) despite this pathogen being highly infectious and widely recognized as a high‐risk zoonosis in abattoirs globally (Gear et al. 1950; De Boni et al. 2022). The frequency of *C. burnetii* infection in individuals employed in high‐risk professions, including farmers, veterinarians and butchers, can vary from 30% to 70% (Aitken et al. 1987; Cook et al. 2021). Approximately 60% of *C. burnetii* infections in people are reported with moderate flu‐like symptoms, with sporadic complications including endocarditis, meningitis and atypical pneumonia (Njeru et al. 2016). Domestic ruminants, including cattle, goats and sheep, have been reported as the source of infection for human illness, and diseased animals shed *C. burnetii* in the stool, urine, dairy products and placenta (Guatteo et al. 2011; Menadi et al. 2020). The most common signs of these infections in livestock are mainly abortion, stillbirth and infertility (Khan and Zahoor 2018).

Policy makers have found KAP studies to be helpful in creating strategies and health education initiatives for the prevention of zoonotic illnesses (Xiang et al. 2010). There is currently limited literature on the seroprevalence, knowledge, attitude and behavioural risk factors of abattoir workers regarding zoonotic illnesses. This study aimed to evaluate the knowledge and practices of abattoir workers in the Eastern Cape Province, SA, regarding brucellosis, coxiellosis/Q‐fever and toxoplasmosis; determine *C. burnetii* seropositivity among abattoir workers in Gauteng Province, SA; and assess the seroprevalence of these diseases among abattoir workers across Africa through a meta‐analysis.

## 2 Materials and Methods

### 2.1 Ethical Approval

Ethical clearance for this study was provided by the Animal and Human Research Ethics Committees. We obtained approval for the research from the Department of Agriculture, Land Reform and Rural Development, under Section 20 of the Animal Diseases Act 1984, Republic of South Africa. Written informed consents were obtained from the abattoir managers, and the recruited abattoir workers provided written informed consent to participate in the study. Ethical clearance for the human blood samples was obtained from the University of Pretoria Faculty of Veterinary Science Research Ethics Committee (V089‐16) and the University of Pretoria Faculty of Health Sciences Research Ethics Committee (519/2017).

### 2.2 Study Area and Study Design

This abattoir‐based study was conducted in three parts: questionnaire interviews on the knowledge and practices regarding the selected zoonotic diseases, a seropositivity study on *C. burnetii* in humans, and a meta‐analysis on three zoonoses in Africa.

The knowledge, practices and exposure questionnaire interviews on selected zoonotic diseases (brucellosis, coxiellosis and toxoplasmosis) were conducted among abattoir workers in the Eastern Cape Province. The Eastern Cape Province has about 16% of the country's livestock (cattle, sheep and goats), totalling 6,256,000 (Zantsi and Nkunjana 2021). We adapted a semi‐structured questionnaire from a similar study on brucellosis, previously conducted in Gauteng Province, South Africa (Kolo et al. 2024). A total of 76 workers from five abattoirs, including butchers, managers, cleaners and meat inspectors, consented to participate in the study. There are 78 red meat abattoirs in the Eastern Cape Province, with an average of 20 and 4–8 workers in high‐throughput and low‐throughput abattoirs, respectively. Therefore, about 90% of the workers in each selected abattoir were sampled. The study could only include workers who were willing to participate in the study. Due to the inability to obtain human ethics approval, we could not collect human blood samples from the workers at the five abattoirs in the Eastern Cape. However, previous studies reported on the seropositivity and molecular characterization of the selected zoonotic infections from livestock samples collected from the same abattoirs where the interviews were conducted (Mazwi et al. 2023; Mazwi et al. 2024).

The *C. burnetii* seropositivity was determined using retrospective human blood samples (*n* = 92) previously collected from workers in six abattoirs (three high‐throughput, three low‐throughput) in Gauteng Province in a study that compared the performance of three *Brucella* spp. serological tests (Kolo et al. 2024). These were the six abattoirs in which slaughtered cattle and sheep were found positive for *Brucella melitensis* and *Brucella abortus* out of 14 abattoirs studied in the province (Kolo et al. 2019), representing 38.9% of the 36 officially registered red meat abattoirs in Gauteng. There is an average of 20 workers per high‐throughput abattoir and 4–8 workers per low‐throughput abattoir; therefore, in each of the six selected abattoirs, about 90% of workers were sampled. The number of samples analysed was based on availability from the previous study, and the inclusion criteria were being active in the meat industry and working in the abattoir industry. Participants included managers and general workers (cleaners, meat inspectors and slaughtermen/butchers).

The meta‐analysis focused on evaluating the seroprevalence of toxoplasmosis, Q‐fever, and brucellosis among abattoir and slaughterhouse workers across various studies conducted in Africa, without considering the year of publication.

### 2.3 Questionnaire Interviews

The questionnaire interviews on knowledge and practices about zoonotic diseases were presented in English by the principal investigator; however, a translator was involved for abattoir workers who did not understand English. Respondents were classified by the type of duties they performed, including butcher (‘slaughterman’), inspector, and others (those engaged in any other type of work conducted at the abattoir). The questionnaire comprised three sections: first, the demographics (age, sex and years at work), second, the knowledge (yes, no) about the three zoonotic diseases and third, the practices that may expose to infection. The questions on practices were: (i) do you take care of animals at home or work on the farm (yes, no); (ii) do you consume unpasteurized milk (yes, no); (iii) do consume uncooked or under‐cooked meat (yes, no); (iv) do you slaughter animals at home (yes, no); (v) do you utilize PPE at work (yes, no). For each indicated practice, a score of +1 was assigned to a correct response, and 0 for an uncertain response or for an incorrect response. A response was incorrect if it was a predisposing factor for zoonotic infections and correct if it reduced the chances of exposure. The scores for the practice questions were pooled to generate a median score. The median score was presented as a binary variable, where median = 1 was regarded as correct with regard to control of zoonotic disease and a median = 0 was regarded as incorrect. The overall practice score was transformed into a binary dependent variable (good or poor practice).

### 2.4 *C. burnetii* Seropositivity

The *C. burnetii* indirect enzyme‐linked immunoassay (iELISA) was conducted on serum specimens from abattoir workers. The ELISA assay was performed at the National Institute for Communicable Diseases (NICD), South Africa, using a commercially available kit (*C. burnetii* ELISA IgG & ELISA IgM, Vircell, S.L. Parque Technológico de la Salud, Avicena 8, 18016 Granada, Spain) according to the manufacturer's instructions.

### 2.5 Seroprevalence Metadata

The meta‐analysis on the seroprevalence of toxoplasmosis, Q‐fever, and brucellosis among abattoir workers/slaughterhouses in Africa followed the Preferred Reporting Items for Systematic Reviews and Meta‐Analyses (PRISMA) guidelines (Shamseer et al. 2015; Page et al. 2021). The search was conducted in two databases: PubMed and Google Scholar. The inclusion criteria for this meta‐analysis were only published peer‐reviewed articles in English and concerning abattoir workers in Africa, regardless of the year published. The search and screening of the studies was performed in November 2023. We did not include grey literature and experimental studies. The search strategies combined search terms referring to the respective disease, pathogen, study subjects, study setting (abattoir) and study area (African countries as follows: ((Abattoir worker OR Abattoir) AND (Coxiella OR Q fever OR Q‐fever OR Brucella or Brucellosis OR Toxoplasma OR Toxoplasmosis) AND (Seroprevalence OR Prevalence) AND (Africa OR Algeria OR Angola OR Benin OR Botswana OR Burkina Faso OR Burundi OR Cameroon OR Cabo Verde OR Cape Verde OR Republic of Cabo Verde OR Central African Republic OR Chad OR Comoros OR Congo OR Republic of the Congo OR Congo‐Brazzaville OR Congo Republic OR DR Congo OR Democratic Republic of Congo OR Zaire OR Côte d'Ivoire OR Ivory Coast OR Djibouti OR Equatorial Guinea OR Egypt OR Eritrea OR Ethiopia OR Gabon OR Gambia OR Ghana OR Guinea OR Guinea‐Bissau OR Kenya OR Lesotho OR Liberia OR Libya OR Madagascar OR Malawi OR Mali OR Mauritania OR Mauritius OR Morocco OR Mozambique OR Namibia OR Niger OR Nigeria OR Rwanda OR Sao Tome and Principe OR Sâo Tomé and Príncipe OR Senegal OR Seychelles OR Sierra Leone OR Somalia OR Somaliland OR Puntland OR South Africa OR South Sudan OR Sudan OR Swaziland OR Eswatini OR Tanzania OR United Republic of Tanzania OR Zanzibar OR Togo OR Tunisia OR Uganda OR Western Sahara OR Zambia OR Zimbabwe)).

**FIGURE 1:**
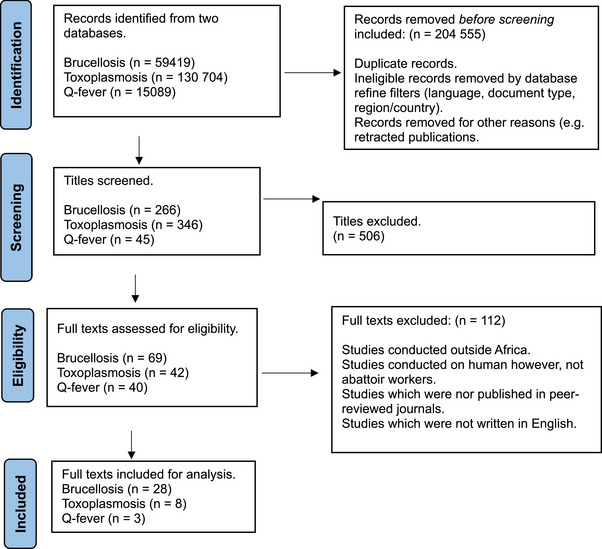
Search strategy for brucellosis, toxoplasmosis and Q‐fever seroprevalence studies among abattoir workers in Africa from Google Scholar and PubMed database.

First, the retrieved titles and abstracts were evaluated by two reviewers (KDM and HvH) following the inclusion/exclusion criteria, followed by identification and removal of duplicate studies (Figure 1). The excluded studies included: studies conducted outside Africa, studies conducted on humans, however, not abattoir workers, studies which were not published in peer‐reviewed journals and studies which were not written in English. One reviewer (KDM) downloaded the full texts of all eligible studies. The full texts were assessed for eligibility by the two reviewers, who then independently extracted relevant data for the study. Any inconsistency between the two reviewers with regard to the retrieved studies and eligibility was resolved through discussion to reach a consensus. Relevant data were extracted into Microsoft Excel sheets, and this included author names, journal, title of study, year of study, number of human serum samples, number of positive cases, the tests used and the country of origin. The extracted data were exported to the R statistical software for analysis.

### 2.6 Data Analysis

#### 2.6.1 Abattoir Workers’ Knowledge

The knowledge response (yes, no) for each of the three zoonotic diseases was assessed for association with each of the six‐abattoir workers' socio‐demographic variables, namely abattoir throughput (low, high), education (tertiary, secondary and none/primary), gender, age (21–30 and 31–60 years), job description (inspector, butcher and other) and years worked at abattoirs (≥3 years, 2 years and 1 year), Only variables with *p* ≤ 0.05 from univariate analyses were combined in a multivariable logistic regression model to establish their association with knowledge about brucellosis, toxoplasmosis, or Q‐fever. Univariate analyses were conducted using chi‐square or Fisher's exact tests, while multivariable analyses employed a stepwise backward elimination procedure with Akaike Information Criteria to establish the most suitable models.

#### 2.6.2 Abattoir Workers’ Practices

The participant score (as transformed binary variable) for all three zoonotic diseases was assessed for association with participant demographic and occupational factors, as indicated for the knowledge section above in univariate and multivariable models.

#### 2.6.3 Human Q‐Fever Seroprevalence

Three explanatory variables (abattoir throughput, sex and age) were each evaluated for association with the occurrence of anti‐*Coxiella* antibodies in univariate analyses. Only variables with *p* ≤ 0.05 were assessed in the final multivariable model to establish the risk factors for *C. burnetii* exposure.

#### 2.6.4 Meta‐Analysis

The estimated pooled prevalence and its 95% confidence intervals for brucellosis, toxoplasmosis and Q‐fever were determined using the random‐effects model, with application of the restricted maximum likelihood method (REML) (Raudenbush and Bryk 1985). Transformation of the data to a normal distribution was performed using the double‐arcsine transformation (PFT) method (Miller 1978). The Cochran's *Q* statistic (Cochran 1954) and the inconsistency index *I* ^2^ (Higgins et al. 2003) were used to test and quantify the variation across studies. Heterogeneity across studies was assessed based on the criteria that values of 25%, 50% and 75% represented low, moderate and high heterogeneity, respectively (Higgins and Thompson 2002). The amount of heterogeneity was also estimated using the true between‐study variance, *τ* ^2^ (Borenstein et al. 2017). Forest plots were generated to visualise heterogeneity across studies on brucellosis, toxoplasmosis and Q‐fever among abattoir workers in Africa. We compared the prevalence of each of the three diseases by geographical region (Western, Eastern, Southern, Northern and Central Africa) and additionally by diagnostic test order for brucellosis, as different techniques are currently being used for the sero‐detection of *Brucella* spp. In records that reported multiple seroprevalences from more than one laboratory method on the same group of people, each value was recorded as a separate study. Data were analysed using the packages ‘metafor’ (Viechtbauer 2010), ‘MASS’ and ‘meta’ (Schwarzer 2007) in the R statistical software version 4.3.1 (R Core Team 2023), at 5% significance level.

## 3 Results

### 3.1 Description of Participants

Of the 76 abattoir workers interviewed in the Eastern Cape Province, 71.1% (54/76) were males and 28.9% (22/76) were females. Most were from high‐throughput abattoirs (76.3%, 58/76), while others were from low‐throughput abattoirs (23.7%, 18/76). A high‐throughput abattoir slaughtered more than 20 livestock per species per day, and a low‐throughput abattoir slaughtered less than 20 livestock per species per day. The workers were categorised into three age groups of 21–30 years (32/76, 42.1%), 31–40 years (20/76, 26.3%) and > 40 years (24/76, 31.6%) (Table S1). Of the 92 abattoir workers tested for antibodies against *C. burnetii* in Gauteng Province, 82.6% (76/92) were males and 17.4% (16/92) were females. An increased number of abattoir workers tested were from the high‐throughput abattoirs (59.8%) compared to the low‐throughput abattoirs (40.2%), and more were 31–60 years old (63/92, 68.5%) compared with the younger age category of 18–30 years (29/92, 31.5%).

### 3.2 Knowledge of Abattoir Workers

#### 3.2.1 Brucellosis

Of the 76 interviewed participants, about half, 36 (47.4%: 95% CI 35.8–59.2), knew about brucellosis. Three variables: abattoir throughput, education and job description were significantly associated (*p* ≤ 0.05) with knowledge about brucellosis (Table S2), and after a backward stepwise elimination procedure, only job description was retained in the final multivariable logistic regression model, showing that knowledge about brucellosis was most frequent among meat inspectors (96%, 24/25), and it was least among other job categories (20.0%, 5/25) (Table 1).

**TABLE 1:** Final multivariable logistic regression model for association between knowledge about brucellosis and abattoir worker demographic or occupational factors.

Abattoir worker factors	Level	No. and % of abattoir workers associated with knowledge about brucellosis	Odds ratio (95% CI)	*p*‐value
Job description				
	Others (*n* = 25) (ref)	5 (20.0)		
	Butchers (*n* = 26)	7 (26.9)	1.5 (0.4, 5.5)	0.561
	Inspector (*n* = 25)	24 (96.0)	95.9 (10.4, 890.2)	< 0.0001

Abbreviation: CI, confidence interval.

#### 3.2.2 Toxoplasmosis

Only 13 of the 76 interviewed abattoir workers (17.1%, 95% CI 9.4–27.5) knew about toxoplasmosis. Univariate analyses showed that only the job description was significantly associated (*p* < 0.0001) with knowledge about toxoplasmosis (Table S3). Frequency of knowledge about toxoplasmosis was 9.4 times higher among meat inspectors (44%, 11/25) compared to butchers (7.7%, 2/26), while none of the participants in the ‘other’ category knew about toxoplasmosis (20%, 5/25) (Table 2).

**TABLE 2:** Multivariable logistic regression model for association between knowledge about toxoplasmosis and abattoir worker demographic or occupational factors.

Abattoir worker factors	Level	No. and % of abattoir workers associated with knowledge about toxoplasmosis	Odds ratio (95% CI)	*p*‐value
Job description				
	Butcher (*n* = 26) (ref)	2 (7.7)		
	Inspector (*n* = 25)	11 (44.0)	9.4 (1.8, 48.8)	0.0075
	Other (*n* = 25)	0 (0)	< 0.01 (0, Inf)	0.9936

*Note*: Butchers were used as the reference category because no participants in the ‘other’ category were knowleageable about toxoplasmosis

Abbreviation: CI, confidence interval.

#### 3.2.3 Q‐Fever

Only 15 (19.7%, 95% CI 11.5–30.5) of the 76 respondents had knowledge about Q‐fever. Univariate analyses showed that only one variable (job description) was significantly associated (*p* = 0.031) with knowledge of Q‐fever among the abattoir workers (Table S4). Inspectors were most frequently knowledgeable about Q‐fever (32%, 8/25), followed by butchers (23.1%, 6/26), while only one of the 25 abattoir workers in the ‘other’ category knew about Q‐fever (Table 3).

**TABLE 3:** Multivariable logistic regression model for association between knowledge about Q‐fever and abattoir worker demographic or occupational factors.

Abattoir worker factors	Level	No. and % of abattoir workers associated with knowledge about fever	Odds ratio (95% CI)	*p*‐value
Job description				
	Other (*n* = 25) (ref)	1 (4.0)		
	Butcher (*n* = 26)	6 (23.1)	7.2 (0.8, 64.9)	0.0784
	Inspector(*n* = 25)	8 (32.0)	11.3 (1.3, 98.8)	0.0285

### 3.3 Practices

Overall, the frequency of good practices against zoonotic diseases was high (59/76, 77.6%, 95% CI 66.6–86.4). The practices considered were rearing of animals at home, consumption of pasteurized milk, consumption of well‐cooked meat that is sourced from abattoirs rather than slaughtered at home, and the wearing of personal protective equipment at work. None of the six socio‐demographic variables analysed was significantly associated with the practices in univariate analyses (p > 0.05) (Table S1).

### 3.4 Seropositivity for *C. burnetii*

Of the 92 abattoir workers' samples with valid test results, 57/92 (61.96%) had *C. burnetii* IgG antibodies (95% CI: 51.3–71.9), which indicates a probable chronic phase or past exposure. Only one abattoir worker (1/92, 1.1%) had IgM antibodies, but with co‐presence of IgG, indicating the acute phase of the infection (2–8 weeks).

The detection of IgG was slightly higher in male workers (63.2%, 48/76) than female (56.3%, 9/16) (Table 4), while the sole participant with IgM antibodies was male (1/76, 1.3%) in the 31–60 age category and from a high‐throughput abattoir. Among the 92 workers tested, IgG antibodies were detected in 44.8% (13/29) of young adults (18–30 years) and in 69.8% (44/63) of older adults (31–60 years). The IgG seropositivity was lower in high‐throughput abattoirs (58.2%, 32/55) than in low‐throughput abattoirs (67.6%, 25/37).

**TABLE 4:** Descriptive and univariate analyses for association between *Coxiella burnetii* seropositivity (IgG) and explanatory variables among abattoir workers.

Variable	Level	No. and % of seropositive abattoir workers	*p*‐value
Abattoir throughput			0.3632
	High (*n* = 55)	32(58.2)	
	Low (*n* = 37)	25(67.6)	
Sex			0.605
	Female (*n* = 16)	9(56.3)	
	Male (*n* = 76)	48(63.2)	
Age (years)			0.022
	18 to 30 (*n* = 29)	13(44.8)	
	31 to 60 (*n* = 63)	44(69.8)	

Univariate analyses showed that only age was significantly associated with *C. burnetii* IgG seropositivity (*p* = 0.022) (Table 4). Older abattoir workers (31–60 years) were significantly more likely (OR = 2.9, *p* = 0.024) to be seropositive for *C. burnetii* than younger workers (18–30 years) (Table 5).

**TABLE 5:** Final multivariable logistic regression model to determine risk factors for *Coxiella burnetii* exposure (IgG antibodies) among abattoir workers.

Variable	Level	Odds ratio (95% CI)	*p*‐value
Age (years)			
	18–30		
	31–60	2.85 (1.15, 7.01)	0.024

### 3.5 Meta‐Analyses

#### 3.5.1 Brucellosis

**FIGURE 2:**
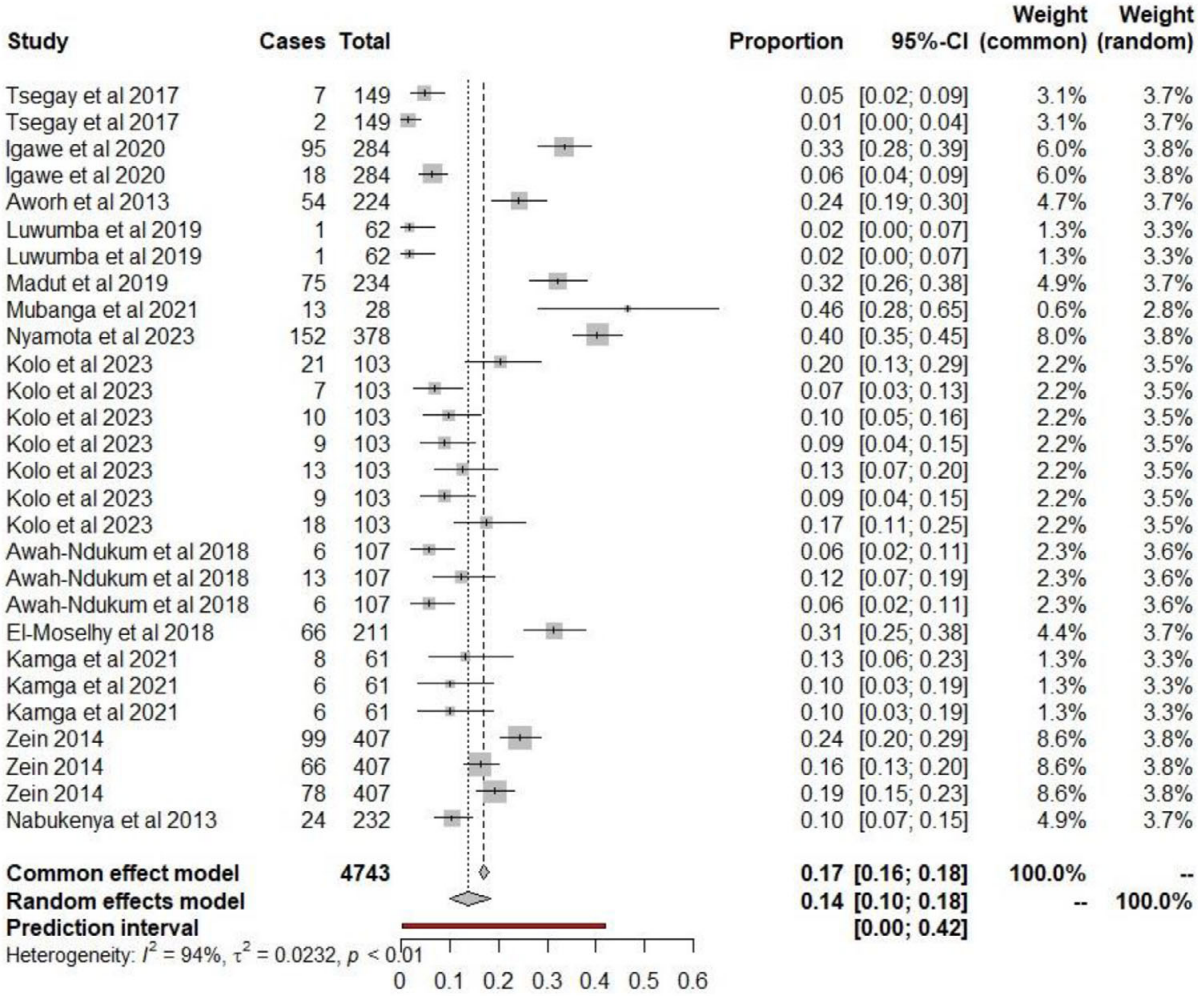
Forest plot of brucellosis seropositivity among abattoir workers in Africa. Number of studies = 28. Number of sampled individuals = 4743. Number of positive cases = 883, *Q* statistic = 449.37, *p* < 0.0001.

A total of 59,300 studies were identified in the initial search on PubMed (119) and Google Scholar (59,300) for the seroprevalence of brucellosis among abattoir workers in Africa (Figure 1). After removing duplicate articles and screening for articles with regard to the selection criteria, 69 full‐text articles (22 PubMed and 47 Google Scholar) were identified. Following the final screening, 13 brucellosis seroprevalence records (28 studies) based on various techniques met the inclusion criteria (Table 6). The estimated pooled prevalence for *Brucella* spp. was 13.8% (95% CI 9.9, 18.2), with a prediction interval of 0%–42.0% (Figure 2). Heterogeneity was high among studies, with *I* ^2^ value of 94.4%, a *Q* statistic of 449.5 (*p* < 0.0001), true and between‐study variance, *τ* ^2^ of 0.0232. The seroprevalence ranged from 1.3% (2/149) from a study in Ethiopia to 46.4% (13/28) from a study in Zambia (Figure 2). The highest brucellosis screening was reported in northern African countries, with a total of 852 abattoir workers from three studies, and the least number of workers screened was in southern Africa (*n* = 103) from one study.

**TABLE 6:** Occurrence of brucellosis among abattoir workers in different regions of Africa.

			No. of abattoir workers tested		
Variable	Level	No. of studies	No. tested	No. positive	% (95% CI)
Brucellosis					
	Central Africa	7	532	58	12.2 (5.5, 20.9)
	Eastern Africa	6	1032	187	7.7 (2.4, 15.3)
	Northern Africa	5	1666	384	24.3 (14.3, 35.8)
	Southern Africa	7	721	87	11.7 (5.4, 19.9)
	Western Africa	3	792	167	19.8 (8.5, 34.3)

#### 3.5.2 Toxoplasmosis

**FIGURE 3:**
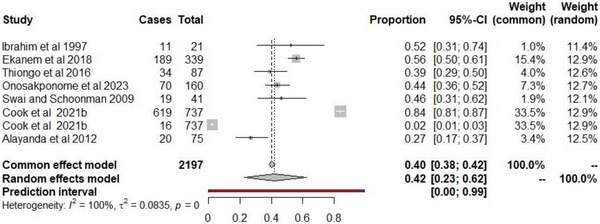
Forest plot of toxoplasmosis seropositivity among abattoir workers in Africa. Number of studies = 8. Number of sampled individuals = 2197. Number of positive cases = 978. *Q* statistic = 1548.05, *p* < 0.0001.

During the first search of the PubMed (10,707) and Google Scholar (120,000) databases for the seroprevalence of toxoplasmosis, a total of 130,707 articles were found (Figure 1). Forty‐two full‐text papers (4 PubMed and 38 Google Scholar) were found after excluding articles that did not meet the selection criteria and eliminating duplicate articles. After the last screening, seven records (eight studies) on the seroprevalence of toxoplasmosis based on techniques among abattoir workers in Africa met the inclusion requirements (Figure 1). This included studies from Egypt, Nigeria, Tanzania and Kenya. The estimated pooled prevalence was 42.0% (95% CI 22.8, 62.5), with a prediction interval of 0.0%–99.2%. Heterogeneity was high (*I* ^2^ = 100, *Q* = 1548, *p *< 0.0001, *τ* ^2 = ^0.0835) (Figure 3). The range of seroprevalence was from 2.2% (16/737) to 83.9% (619/737), with both limits reported in Kenya (Figure 3).

#### 3.5.3 Q‐Fever

**FIGURE 4:**
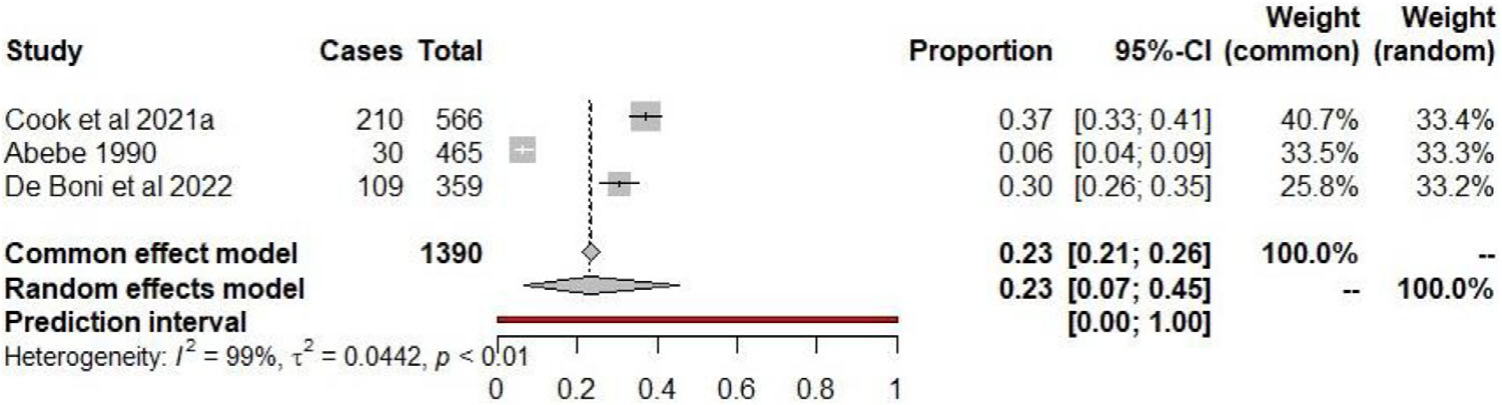
Forest plot of Q‐fever seropositivity among abattoir workers in Africa. Number of studies = 3. Number of sampled individuals = 1390. Number of positive cases = 349. *Q* statistic = 173.07, *p* < 0.0001.

A total of 15 089 articles were identified in the initial search of PubMed (89) and Google Scholar (15 000) databases for the seroprevalence of Q‐fever (Figure 1). After removing duplicate articles and screening for articles that met the selection criteria, 45 full‐text articles (8 PubMed and 37 Google Scholar) were identified. Following the final screening, three Q‐fever seroprevalence studies among abattoir workers in Africa met the inclusion criteria. This included studies from Kenya, Ethiopia and South Africa. The estimated pooled prevalence was 22.9% (95% CI 6.5, 45.3) and a prediction interval of 0 to 100%. Heterogeneity was high (*I* ^2 = ^98.8%, *Q* = 173.1, *p *< 0.0001, *τ* ^2 = ^0.0442) (Figure 4), with a reported range of 6.5% (30/465) to 37.1% (210/566) reported from Ethiopia and Kenya, respectively (Figure 4). Only two African regions reported the seroprevalence of Q‐fever from three studies.

## 4 Discussion

In abattoirs, workers have daily direct contact with livestock, and they are likely to face increased exposure to zoonotic diseases. This study identified a gap in knowledge and practices regarding the selected diseases (brucellosis, coxiellosis/Q‐fever and toxoplasmosis) among Eastern Cape Province abattoir workers who participated in the questionnaire. The study indicated abattoir throughput, job description and education had a significant association (*p* ≤ 0.05), with knowledge of brucellosis, while only job description had a significant association (*p* ≤ 0.05) with knowledge of toxoplasmosis. Furthermore, this study reports the sero‐positivity of *C. burnetii* IgG antibodies in 61.9% and IgM antibodies in 1.1% of the Gauteng abattoir workers. The IgG antibodies mainly indicate the chronic phase of infection, while the presence of IgM and IgG antibodies indicates the acute phase of infection (Parker et al. 2006). The study reports a pooled prevalence among abattoir workers in Africa of 13.8% in brucellosis, 42.0% in toxoplasmosis and 22.9% in Q‐fever.

Knowledge, attitudes, risk factors and practices surveys are a popular method for gathering data to evaluate safe work practices in populations that are at risk (Rahim et al. 2012). Of the 76 abattoir workers, the knowledge for brucellosis, toxoplasmosis and coxiellosis/Q‐fever was reported in 47.4%, 17.1% and 19.7%, respectively. A recent study conducted in Rwanda among abattoir workers reported that 27.1% and 8.5% were familiar with brucellosis and Q‐fever, respectively (Ntivuguruzwa et al. 2021). The increased knowledge among the workers in our study area on brucellosis may be due to recent training which were reported. Our findings on the knowledge of brucellosis are in agreement with a study conducted in Ethiopia (44.2%) (Tsegay et al. 2017), a study conducted in Tanzania (76%) reported a higher knowledge proportion among the workers (Denice et al. 2019). On the contrary, Özlü et al. (2020) reported 8.0% on toxoplasmosis knowledge among cattle farmers; this could be due to the lack of frequent informative sessions and continuous routine diagnosis.

It is imperative to underline the consequences of occupational exposure to zoonotic diseases in abattoir workers, precisely through inhalation of contaminated aerosols and direct contact with infected slaughtered livestock. Recently, a study conducted in Eastern Cape abattoirs reported the serological evidence of brucellosis, coxiellosis and toxoplasmosis in livestock (Mazwi et al. 2023). In this study, abattoir workers from high‐throughput abattoirs were more frequently knowledgeable about brucellosis 55.1% than those from low‐throughput abattoirs 22.2%, although this was not statistically significant. It is evident by the univariate analyses that job description and education had a significant association (*p* ≤ 0.05) with knowledge on toxoplasmosis and brucellosis, respectively, among the abattoir workers. Similar studies conducted on zoonotic diseases emphasized that education makes a substantial contribution to practices and knowledge (Lindahl et al. 2015; Al‐Shamahy et al. 2000). Thus, health education regarding zoonotic diseases among communities could be implemented as part of the disease control programs (Lindahl et al. 2015; Smits 2012).

Of the 76 abattoir workers, 31.6%, 10.5%, 86.8% practiced consumption of unpasteurised milk, consumption of uncooked/undercooked meat and wearing personal protective equipment, respectively. The reported consumption of unpasteurised milk and uncooked/undercooked meat increases the risk of zoonotic infections among the workers. Regarding the slaughtering procedure, 61.8% of the abattoir workers practised home slaughtering, which included cattle, goats, sheep and pigs. Slaughtering brucellosis‐infected livestock has been reported as a high‐risk activity, given that handling infected meat and conducting the slaughter may expose those involved if safety measures are not implemented (Galinska and Zagórski 2013).

Human Q‐fever cases are likely underreported, and accurate diagnosis depends primarily on a healthcare provider's knowledge of the illness's symptoms and the availability of a dependable diagnostic laboratory (Fournier et al. 1998). The *C. burnetii* ELISA test is less specific and usually results in false positives due to the presence of *Coxiella*‐like bacteria in samples (Guimard et al. 2017). It has been established that *Coxiella*‐like bacteria are a novel human infectious agent, which may explain the reported increased sero‐positivity of *C. burnetii* observed in humans (Guimard et al. 2017; Rahal et al. 2020).

A recent study conducted in South Africa reported an overall IgG antibody seropositivity of *C. burnetii* (33%) from abattoir workers (De Boni et al. 2022). An increased IgG exposure was observed in this study from males (63.2%) as compared to female (56.3%) abattoir workers. A similar study conducted in Western Kenya identified male workers as a risk factor for *C. burnetii* seropositivity as compared to female workers (Cook et al. 2021). Our findings, although not statistically significant, are in line with other research showing that male employees are more likely to test positive for *C. burnetii* (Cook et al. 2021; Carrieri et al. 2002). This finding could be explained by the fact that men perform higher‐risk responsibilities in the slaughterhouse, such as slaughtering and eviscerating animals. In our study, the age group between 31–60 reported the positivity of 69.8% IgG and 1.6% IgM as compared to those between the age of 18–30 years (44.8% IgG). The IgM‐positive male abattoir worker was from a high‐throughput abattoir. Older abattoir workers of 31–60 years were significantly more likely (OR = 2.9, *p* = 0.024) to be seropositive for *C. burnetii* than younger workers (18–30 years of age). The increased positivity in older workers may be due to a compromised immune system or the number of years spent working in the abattoirs, thus increasing the risks of exposure.

The results of the meta‐analysis seroprevalence studies showed that the infections (brucellosis, toxoplasmosis & Q‐fever) are present among abattoir workers in Africa; however, they may be under‐reported as shown by the limited published studies. The results of the brucellosis meta‐analyses in African abattoir workers stratified by regions indicated the lowest seroprevalence among Eastern African countries, 7.7% (Cl:2.4–15.3), and the highest among the northern African countries, 24.3% (Cl:14.3–35.8). Based on observation, a reduced number of studies have been conducted in Africa on toxoplasmosis and Q‐fever among abattoir workers. This study evaluated eight studies conducted in Africa on toxoplasmosis and only three studies on Q‐fever infection among abattoir workers. The seropositivity of toxoplasmosis ranged between 39.7%–52.4% from the eastern African countries to Western African countries, respectively. The seroprevalence of Q‐fever was 19.4% and 30.4% reported from eastern and southern Africa, respectively. A recent meta‐analysis study conducted in Australia reported the Q‐fever seroprevalence between 4.7% and 91.7% among abattoir and slaughterhouse workers (Woldeyohannes et al. 2018). Their study indicated that slaughtering of livestock, including cattle, sheep and goats, had the most significant risk factors associated with seropositivity (Woldeyohannes et al. 2018), which could account for the high seropositivity observed in Gauteng abattoir workers that needs further investigation.

## 5 Conclusion

This study showed that there is a gap in the knowledge and practices of zoonotic diseases among abattoir workers in the studied abattoirs. Lack of knowledge observed regarding the selected infections raises a concern, as abattoir workers have high exposure potential to zoonotic infections. The seropositivity of toxoplasmosis, Q‐fever and brucellosis among abattoir workers has been reported across Africa. It is strongly advised that employees in high‐risk industries participate in vaccination programs to reduce the risk of acquiring zoonotic infections and the associated chronic illnesses.

## 6 Limitations and Recommendations

Only 76 abattoir workers consented to participate in the study from the five abattoirs in Eastern Cape Province, and only 92 human serum samples were analysed for *C*. *burnetii* seropositivity, as decided by availability from a previous study in Gauteng Province. Most of the low‐throughput abattoirs have fewer workers, ranging from 4 to 8 employees, as they only slaughter a few animals per day. Abattoir workers who were not keen to participate in the study were excluded, hence the low number. Based on the outcomes of this study, open‐ended questionnaires should be used for abattoir research as this allows the workers to share their knowledge and understanding in detail, thus allowing better interpretations. There is a need for further studies that explore more variables for seropositivity, such as behaviour regarding consumption of animal products and household environmental settings. The only retained variable in multivariable analysis (age) showed goodness‐of‐fit for the data (Hosmer–Lemeshow goodness‐of‐fit *p* = 0.279), but inclusion of other variables could provide a better explanation of the seropositivity model and a more comprehensive interpretation of the risk factors for *C*. *burnetii* seropositivity.

## Author Contributions

**K. D. Mazwi**: investigation, writing – original draft, methodology, writing – review and editing, conceptualization. **C. Byaruhanga**: writing – original draft, writing – review and editing, formal analysis. **H. Geyer**: methodology, investigation. **F. B. Kolo**: conceptualization, supervision, methodology. **I. F. Jaja**: conceptualization, methodology, supervision. **S. O. Ochai**: investigation, methodology. **H. van Heerden**: conceptualization, funding acquisition, writing – review and editing, visualization, validation, project administration, supervision, resources.

## Funding

We would like to appreciate the Department of Veterinary Tropical Diseases (AgriSETA), South Africa, the Belgian Directorate‐General for Development Cooperation (DGD) within the DGD‐ITM Framework Agreement 4 and 5, the Centers for Disease Control and Prevention and UNICEF for the research funding.

## Ethics Statement

Ethical clearance for this study was provided by the Animal (REC028‐22) and Human (HUM040/0821) Research Ethics Committees of the University of Pretoria, South Africa. We obtained approval for the research from the Department of Agriculture, Land Reform and Rural Development, under Section 20 of the Animal Diseases Act 1984, Republic of South Africa (reference number). Written informed consent was obtained from the abattoir managers and the recruited abattoir worker participants for their voluntary participation in the study. Ethical clearance for the human blood samples was obtained from the University of Pretoria Faculty of Veterinary Science Research Ethics Committee (V089‐16) and the University of Pretoria Faculty of Health Sciences Research Ethics Committee (519/2017).

## Conflicts of Interest

The authors declare no conflicts of interest.

## Supporting information




**Supplementary Table 1**. A descriptive and univariate assessment of the association between abattoir workers’ demographic or occupational factors and practices regarding zoonotic diseases
**Supplementary Table 2**. A descriptive and univariate assessment for the association between abattoir workers’ knowledge on brucellosis and demographic or occupational factors
**Supplementary Table 3**. A descriptive and univariate assessment for the association between abattoir workers’ knowledge about toxoplasmosis and demographic or occupational factors
**Supplementary Table 4**. A descriptive and univariate assessment for the association between abattoir workers’ knowledge about Q fever and demographic or occupational factors

## Data Availability

Supplementary data has been submitted.
