# Supplementary material for: Awareness of Zoonotic Infections and a Seroprevalence Meta‐Analysis of Brucellosis, Q‐Fever and Toxoplasmosis Among Abattoir Workers
Source: Vet Med Sci. 2026 May 14;12(3):e70985. doi: 10.1002/vms3.70985 (PMC13172768; doi:10.1002/vms3.70985)
Supplement: Supplementary file 1 — Supplementary Table 1. A descriptive and univariate assessment of the association between abattoir workers’ demographic or occupational factors and practices regarding zoonotic diseases Supplementary Table 2. A descriptive and univariate assessment for the association between abattoir workers’ knowledge on brucellosis and demographic or occupational factors Supplementary Table 3. A descriptive and univariate assessment for the association between abattoir workers’ knowledge about toxoplasmosis and demographic or occupational factors Supplementary Table 4. A descriptive and univariate assessment for the association between abattoir workers’ knowledge about Q fever and demographic or occupational factors [file VMS3-12-e70985-s001.docx]

**Supplementary Table 1.** A descriptive and univariate assessment of the association between abattoir workers’ demographic or occupational factors and practices regarding zoonotic diseases

| **Abattoir worker factors** | **Level** | **No. and % of abattoir workers with predisposing practices** | **p-value** |
| --- | --- | --- | --- |
| Abattoir throughput |  |  | 0.7474 |
|  | Low(n=18) | 3(16.7) |  |
|  | High(n=58) | 14(24.1) |  |
| Education |  |  | 0.5199 |
|  | None or primary(n=10) | 3(30.0) |  |
|  | Secondary(n=27) | 4(14.8) |  |
|  | Tertiary(n=15) | 3(20.0) |  |
| Age (years) |  |  | 0.1971 |
|  | 21 to 30(n=32) | 10(31.3) |  |
|  | 31 to 40 (n=20) | 2(10.0) |  |
|  | >40(n=24) | 5(20.8) |  |
| Gender |  |  | 0.1272 |
|  | Female(n=22) | 2(9.1) |  |
|  | Male(n=54) | 15(27.8) |  |
| Job description |  |  | 0.3323 |
|  | ^a^Other(n=25) | 7(28.0) |  |
|  | Butcher(n=26) | 7(26.9) |  |
|  | Inspector(n=25) | 3(12.0) |  |
| Years of work at abattoir |  |  | 0.5735 |
|  | One year (n=25) | 6(24.0) |  |
|  | Two years (n=13) | 4(30.8) |  |
|  | ≥Three years (n=38) | 7(18.4) |  |

^a^Others includes transporters. The zoonotic diseases considered were brucellosis, toxoplasmosis and Q-fever.

**Supplementary Table 2.** A descriptive and univariate assessment for the association between abattoir workers’ knowledge on brucellosis and demographic or occupational factors

| **Abattoir worker factors** | **Level** | **No. and % of abattoir workers with knowledge about brucellosis** | **p-value** |
| --- | --- | --- | --- |
| Abattoir throughput |  |  | 0.0168 |
|  | Low(n=18) | 4(22.2) |  |
|  | High(n=58) | 32(55.1) |  |
| Education |  |  | 0.00985 |
|  | None or primary(n=10) | 3(30.0) |  |
|  | Secondary(n=27) | 14(51.9) |  |
|  | Tertiary(n=15) | 13(86.7) |  |
|  |  |  |  |
| Age (years) |  |  | 0.6759 |
|  | 21 to 30(n=32) | 15(46.9) |  |
|  | 31 to 40(n=20) | 11(55.0) |  |
|  | >40(n=24) | 10(41.7) |  |
| Gender |  |  | 0.0698 |
|  | Female(n=22) | 14(63.6) |  |
|  | Male(n=54) | 22(40.7) |  |
| Job description |  |  | <0.0001 |
|  | Other(n=25) | 5(20.0) |  |
|  | Butcher(n=26) | 7(26.9) |  |
|  | Inspector(n=25) | 24(96.0) |  |
| Years of work at abattoir |  |  | 0.1828 |
|  | One year(n=25) | 9(36.0) |  |
|  | Two years(n=13) | 5(38.5) |  |
|  | Three and more(n=38) | 22(57.9) |  |

^a^Others includes transporters,…….,…….

**Supplementary Table 3.** A descriptive and univariate assessment for the association between abattoir workers’ knowledge about toxoplasmosis and demographic or occupational factors

| **Abattoir worker factors** | **Level** | **No. and % of abattoir workers with knowledge about toxoplasmosis** | **p-value** |
| --- | --- | --- | --- |
| Abattoir throughput |  |  | 1.0 |
|  | Low(n=18) | 3(16.7) |  |
|  | High(n=58) | 10(17.2) |  |
| Education |  |  | 0.054 |
|  | None or primary(n=10) | 0(0) |  |
|  | Secondary(n=27) | 5(18.5) |  |
|  | Tertiary(n=15) | 6(40.0) |  |
|  |  |  |  |
| Age (years) |  |  | 0.604 |
|  | 21 to 30(n=32) | 6(18.8) |  |
|  | 31 to 40(n=20) | 2(10.0) |  |
|  | >40(n=24) | 5(20.8) |  |
| Gender |  |  | 0.3246 |
|  | Female(n=22) | 2(9.1) |  |
|  | Male(n=54) | 11(20.4) |  |
| Job description |  |  | <0.0001 |
|  | Other(n=25) | 0(0) |  |
|  | Butcher(n=26) | 2(7.7) |  |
|  | Inspector(n=25) | 11(44.0) |  |
|  |  |  |  |
| Years of work at abattoir |  |  | 0.4197 |
|  | One year(n=25) | 3(12.0) |  |
|  | Two years(n=13) | 1(7.7) |  |
|  | Three and more(n=38) | 9(23.7) |  |

^a^Others includes transporters,…….,…….

**Supplementary Table 4.** A descriptive and univariate assessment for the association between abattoir workers’ knowledge about Q fever and demographic or occupational factors

| **Abattoir worker factors** | **Level** | **No. and % of abattoir workers with knowledge about Q fever** | **p-value** |
| --- | --- | --- | --- |
| Abattoir throughput |  |  | 0.3265 |
|  | High(n=58) | 10(17.2) |  |
|  | Low(n=18) | 5(27.8) |  |
| Education |  |  | 0.9141 |
|  | None or primary(n=10) | 3(30.0) |  |
|  | Secondary(n=27) | 6(22.2) |  |
|  | Tertiary(n=15) | 4(26.7) |  |
| Age (years) |  |  | 0.8213 |
|  | 21 to 30(n=32) | 7(21.9) |  |
|  | 31 to 40(n=20) | 3(15.0) |  |
|  | >40(n=24) | 5(20.8) |  |
| Gender |  |  | 0.9141 |
|  | Female(n=22) | 3(13.6) |  |
|  | Male(n=54) | 12(22.2) |  |
| Job description |  |  | 0.0306 |
|  | Other(n=25) | 1(4.0) |  |
|  | Butcher(n=26) | 6(23.1) |  |
|  | Inspector(n=25) | 8(32.0) |  |
| Years of work at abattoir |  |  | 0.5915 |
|  | One year(n=25) | 5(20.0) |  |
|  | Two years(n=13) | 1(7.7) |  |
|  | Three and more(n=38) | 9(23.7) |  |

^a^Others includes transporters,…….,…….
